# Greater genetic diversity is needed in human pluripotent stem cell models

**DOI:** 10.1038/s41467-022-34940-z

**Published:** 2022-11-26

**Authors:** Sulagna Ghosh, Ralda Nehme, Lindy E. Barrett

**Affiliations:** 1 Stanley Center for Psychiatric Research, Broad Institute of MIT and Harvard, Cambridge, MA 02142 USA; 2 Department of Stem Cell and Regenerative Biology, Harvard University, Cambridge, MA 02138 USA

**Keywords:** Pluripotent stem cells, Research data

## Abstract

While there are a growing number of human pluripotent stem cell repositories, genetic diversity remains limited in most collections and studies. Here, we discuss the importance of incorporating diverse ancestries in these models to improve equity and accelerate biological discovery.

## Genetic diversity in genome research and stem cell repositories

**Fig. 1:**
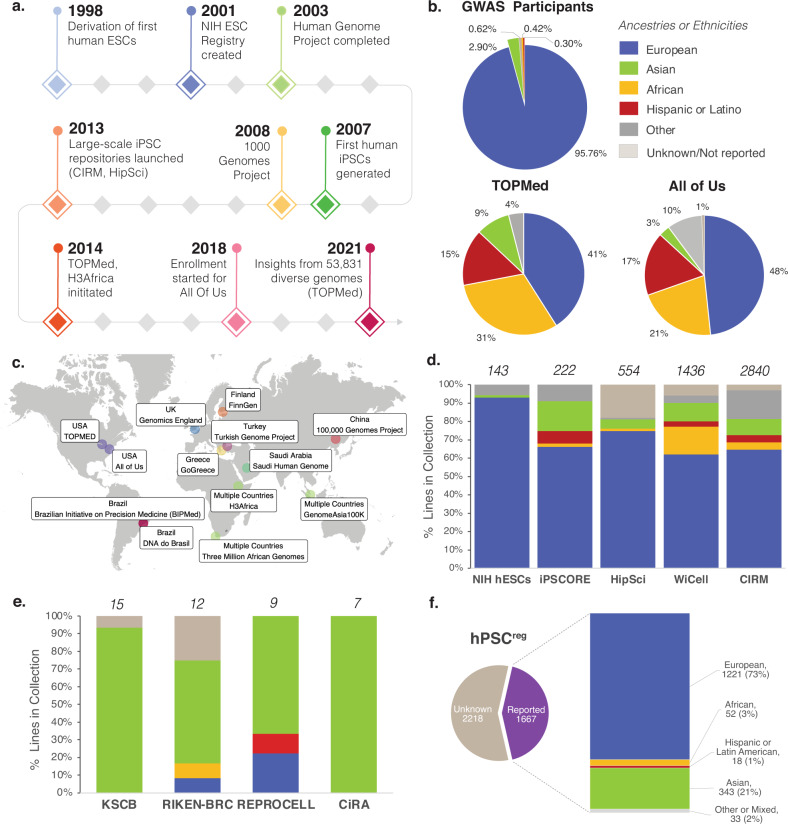
Genetic diversity in genomic research and stem cell repositories. **a** Breakthroughs over the last 20 years in human genomics and stem cell research offer opportunities for understanding how genetic variation shapes disease using scalable in vitro models. **b** Most participants in genome wide associations studies are of European ancestry. Large scale genomics studies such as the Trans-Omics for Precision Medicine (TOPMed) program and the All of Us Research program aim to address this. Genetically inferred ancestry or self-reported race or ethnicity data from each study was grouped into super populations. **c** Several efforts have been launched globally to prioritize inclusion of underrepresented participants in human genomic research. **d** Most pluripotent stem cell lines in large-scale collections are derived from donors of European ancestry. The total number of cell lines in each collection as of 2021 is noted above each bar. Data was taken from the websites of public repositories (“HipSci [https://www.hipsci.org/lines/#/lines]”; “WiCell [https://www.wicell.org/home/stem-cells/catalog-of-stem-cell-lines/advanced-search.cmsx]”; “hESC [https://grants.nih.gov/stem_cells/registry/current.htm?sort=afda]”; “CIRM [https://www.fujifilmcdi.com/search-cirm/]” and personal communication) or a peer-reviewed study (iPSCore)^36^ and grouped into super populations. **e** Additional smaller-scale collections from the National Stem Cell Bank of Korea (including ESCs and iPSCs), unaffected control iPSCs from RIKEN BRC, HLA-homozygous iPSCs from the CiRA Foundation and iPSCs from REPROCELL. The total number of cell lines from independent donors in each collection is noted above each bar. Data was collapsed into supergroups, taken from the websites of the “National Stem Cell Bank of Korea [https://nih.go.kr/contents.es?mid=a50401110300]” and personal communications; “RIKEN BRC [https://cell.brc.riken.jp/en/]”; “CiRA Foundation [https://www.cira-foundation.or.jp/e/research-institution/ips-stock-project/homozygous.html]”; “REPROCELL [https://www.reprocell.com/product-catalog/induced-pluripotent-stem-cells]”. **f** Data from hPSCreg showing the breakdown of cell lines with reported race or ethnicity data collapsed into super populations. Percentages shown are based on lines with reported race or ethnicity. Data were obtained from and processed with support of the “human pluripotent stem cell registry [www.hpscreg.eu]” (EU GA101074135).

Over the past twenty years, the field of human genetics has advanced tremendously (Fig. 1a), providing crucial insights about risk variants and protective alleles underlying a range of complex diseases. Individual genomes can now be sequenced for less than $600^1^ and large population-based biobanks are allowing researchers to directly link genetic profiles to disease phenotypes. Despite these advances, a well-documented issue in existing genomic data is the inadequate representation of samples with non-European ancestries^2^. For instance, in genome wide association studies (GWAS), commonly used to discover connections between disease risk and genomic variants, most participants are of European ancestry (95.76%)^3^. Participants of Asian ancestry collectively represent 2.9%, while participants of African ancestry and participants of Hispanic/Latin American ancestries make up less than 1% (Fig. 1b)^3^. It has become increasingly evident that this lack of genomic diversity misses opportunities to discover effects of variants that are common in underrepresented groups and produces polygenic risk scores that are less effective in these populations^4^. This in turn can exacerbate clinical misdiagnosis and deepen existing health care disparities^2^. To prioritize greater diversity in genomic research, numerous initiatives have been launched worldwide in recent years to incorporate underrepresented populations (Fig. 1c). These genomic databases are beginning to bear fruit and have already led to new insights about rare, population-specific risk variants, differential susceptibility to diseases and variability in drug response across populations. For instance, a recent whole genome sequencing study of 426 participants of African ancestry from 13 African countries (H3 Africa) revealed 3 million previously undiscovered variants with broad relevance for clinical genomics and variant interpretation^5^. Specifically, the study found that certain rare variants suspected to cause disease were quite common in populations of African ancestry suggesting that they might be less pathogenic than previously thought^5^. Similarly, a study of 1739 individuals across 219 population groups in Asia found a higher frequency of genetic variants related to adverse drug response to anti-clotting medications such as Warfarin and Clopidogrel in individuals with North Asian ancestry^6^. Findings from such efforts are not only reshaping our thinking about disease risk prediction and patient stratification, but also paving the way for reducing inequities in health care applications.

As genomic studies diversify, the number of disease-relevant alleles that are differentially enriched across global populations, or that differentially impact populations due to unique genetic or non-genetic modifiers, will likely increase. To understand how these risk variants influence disease etiology and how such effects vary across ancestries, we need experimental models that capture the genetic diversity present in human populations. Human pluripotent stem cells (hPSCs), including embryonic stem cells (ESCs) and induced pluripotent stem cells (iPSCs), can be used to generate such systems as they can be obtained from individuals of diverse genetic backgrounds, differentiated into disease-relevant cell types, and engineered to harbor one or more genetic variants of interest. Recognizing the utility of such cellular models, several large-scale stem cell repositories have emerged worldwide to ensure standardized collection, reprogramming methods, storage, and distribution of thousands of hPSC lines (Fig. 1d). While hPSC banks have enabled large-scale investigations of molecular and cellular traits associated with genotypic variation^7^, there is a striking overrepresentation of donors of European ancestry. For example, a recent whole genome sequencing study revealed that out of 143 human ESCs listed in the National Institutes of Health (NIH) registry, 93% are derived from donors of European ancestry^8^. Similarly, samples from donors of reported European ancestry on average contribute to more than 67% of iPSC lines across multiple larger repositories compared to cell lines derived from donors of reported African ancestry, Asian ancestry, or Hispanic or Latino ethnicities (Fig. 1d). By comparison, on a smaller scale, multiple national banks have emerged which are oriented towards providing health care and have been built on a strategy of most effectively treating the local population based on haplotype (e.g., 14 unique donors of Korean ancestry in the National Stem Cell Bank of Korea and 7 unique human leukocyte antigen (HLA)-homozygous donors of Japanese ancestry from the Center for iPS Cell Research and Application (CiRA) Foundation). Figure 1e illustrates several additional resources including the National Stem Cell Bank of Korea and CiRA Foundation, as well as unaffected control iPSCs from RIKEN BRC and REPROCELL. While these banks are in their early stages and numbers reflect the current composition, based on publicly available data, they complement global efforts and are excellent resources for the given ancestries represented in the collections. Taken together, the ancestry breakdown of available hPSC lines is well captured in “hPSCreg [https://hpscreg.eu/]”, a public registry which catalogs stem cell lines across several banks along with standardized biological and legal information (Fig. 1f). While these resources are continually expanding, a major drawback at present is that experimental models may be biased toward hPSCs from individuals of European ancestry based on cell line accessibility. As in the case of genomics research, this lack of diversity can lead to imbalanced outcomes and missed opportunities for biological discovery if overlooked.

## Incorporating diversity in stem cell models to accelerate research and discovery

In recent years, the use of hPSC-based models has rapidly expanded beyond basic research and has begun to revolutionize the pharmaceutical industry, with applications in drug discovery and toxicity^9^. These advances, however, also underscore the importance of expanding the genetic diversity of stem cell models to ensure equitable, timely, and broadly applicable scientific insights and medical benefits. Genetically diverse cellular models can be important drivers of scientific and therapeutic discovery for several reasons. First, including cell lines from diverse genetic backgrounds in functional studies can facilitate the identification of genetic variants underlying specific traits and disorders. Populations of African ancestry for instance have more genetic variation compared with the rest of the world. African genomes have shorter haploblocks and exhibit less linkage disequilibrium (LD) which allows causal alleles for a particular trait or disorder to be mapped at higher spatial resolution^10,11^. Indeed, despite a relatively small sample size, the H3Africa study^5^ discussed above successfully identified several novel disease-associated variants, a higher number than what is typically discovered in similar studies based on participants of European ancestry. Second, certain disease-associated risk variants may be more or less prevalent in individuals from specific ancestries. For example, a genetic variant that doubles the risk of respiratory failure from COVID-19 occurs in 60% of individuals of South Asian ancestry compared to 15% of individuals of European ancestry^12^. Studying the functional impacts of such variants would therefore benefit from greater access to cell lines generated from donors of South Asian ancestry. As efforts to increase the diversity in genomic studies come to fruition, such examples will only increase. Third, the growing number of iPSC-based clinical trials^13^ highlights their potential in cell replacement therapy. Since autologous transplantation is neither time nor cost effective, efforts have focused instead on the matching of HLA haplotypes to reduce immune rejection. In this approach, iPSC lines are derived from donors homozygous for a few HLA haplotypes common in a given population. For example, a selection of 10 iPSC lines with the most common HLA types in the United Kingdom (UK) would provide a beneficial match for two-thirds of the UK population^14^. As the most common HLA types vary across ancestries, it is critical to ensure that similar efforts include HLA haplotypes representative of additional global populations through either cell collection efforts or gene editing^15^, such as the efforts of the CiRA Foundation in Japan discussed above. Finally, non-genetic modifiers, such as co-infection, microbiomes, diet, and socio-cultural factors may also contribute to phenotypic differences across populations. Some of these effects can be tested in hPSC-based models to disentangle genetic versus non-genetic contributors.

While the extent to which different ancestral backgrounds might impact phenotypes in iPSC-based in vitro models is not well understood, studies suggest that inter-individual genetic changes, including common genetic variation, can result in quantifiable differences in cellular phenotypes. One study of iPSC lines derived from 301 donors found that genetic differences between individual donors affected most cellular traits in iPSCs, including differentiation capacity and cellular morphology^7^. Another study of 110 iPSC lines identified rare deleterious non-synonymous single nucleotide variants (nsSNVs) that were associated with outlier cellular phenotypes, which deviated significantly from modal phenotypic values^16^. Of note, the iPSC lines utilized in both studies were predominantly from donors of European ancestry. Increasing the number of cell lines from donors of additional ancestries are thus needed to enable similarly scaled analyses across populations. In that regard, one study using 72 dermal fibroblast-iPSC pairs from donors of African American ancestry and donors of European ancestry identified ancestry-dependent transcriptional profiles that were associated with reprogramming efficiency^17^. Additionally, a study of iPSC-derived hepatocytes from 28 individuals revealed that donor genetic background was sufficient to drive differences in hepatocyte differentiation propensity across cell lines^18^, highlighting the potential for inter-individual genetic variation to impact cell type-relevant preclinical toxicity measurements. Together, these reports suggest that some genetic differences across individuals from diverse ancestries have the potential to manifest as measurable differences in cellular phenotypes in iPSC-based in vitro models.

From a practical perspective, it would be valuable to reflect upon the approaches and assays for which the inclusion of genetically diverse iPSC lines would be most critical. Here, we highlight three specific examples. First, examining the effect of a particular pharmacological perturbation is crucial. Pharmacological treatment responses can present strong differences across ancestral groups^19^. For example, as discussed above, treatment response to Warfarin shows greater variation in individuals of North Asian ancestry as compared to individuals of European ancestry^6^. This presents a challenge for scientific and pharmaceutical industries, as experimental validation and patient recruitment for clinical trials are predominantly performed in the United States and Europe^13^. While establishing clinical trials across the globe would be costly and time-consuming, iPSCs can be readily generated using samples from ancestrally and geographically diverse donors, which could pave the way for the investigation of pharmacogenetic effects on iPSC-derived cell types to optimize drug treatments. Given the importance of HLA haplotypes discussed above, one study screened HLA haplotypes from 1000 participants in Taiwan, identifying 13 homozygous HLA haplotypes, and subsequently generating iPSC lines from all of them. Cardiomyocytes and neurons derived from the 13 lines were then used in toxicity screens, illustrating the utility of a population-based hPSC approach to assess cytotoxicity^20^. Second, introducing protective or pathogenic variants by gene editing on multiple genetic backgrounds is also critical. Given the potential for modifier alleles to influence phenotypes, evaluating the effects of variants across multiple human genetic backgrounds, rather than a single isogenic background, may generate novel insights. Third, studying the effect of natural genetic variation on cellular phenotypes such as expression quantitative trait loci is important. The availability of large-scale hPSC repositories (Fig. 1) has fueled the expansion of in vitro human population genetics, enabling the identification of genotype-phenotype associations that can be measured across hundreds of genetic variants and traits^21^. Using the example discussed above, since populations of African ancestry harbor more genetic diversity and have shorter range LD compared with other populations^10,11^, using hPSC lines from donors of African ancestry has the potential to uncover more effects and map them at higher resolution. Taken together, such efforts are not only important to avoid exacerbating existing disparities, but also hold promise for novel genetic and translational insights.

## Challenges and opportunities for enabling more equitable cellular resources

While the need for more diverse hPSC resources is clear, substantial challenges remain with regard to expanding cell collections and implementing these resources in the laboratory. First, it is critical to acknowledge that current efforts toward greater inclusivity exist in a historical context of discrimination, where actions as well as inactions have eroded trust in scientific and medical establishments (for additional discussion on historical issues of race and ancestry in medicine, please see ref. 22). Therefore, conscious efforts toward rebuilding trust and increasing participation are essential, including ensuring informed consent; broad access to collected resources, data and results; purposeful and continued engagement with all stakeholders contributing to cell collections; and clear, accurate language to describe race, ethnicity, ancestry, and their potential roles in specific biological findings. As global cell collections expand, it is essential to invest in training and capacity building specifically within currently underrepresented countries, and to establish scientific partnerships to facilitate the utilization of hPSC resources in the communities from which they are derived. In parallel, countries with established cell banking capacities must continue to improve representation of diverse ancestries from donors that may be available within those countries. Cell line distribution and data-sharing can also be subject to country-specific limitations, which underscores the importance of simultaneously advocating for increased diversity of collections within a given country, as well as increasing global collaborative efforts between countries. Data governance is another important consideration. While deeper and more extensive clinical phenotyping and metadata would be extremely valuable when coupled with genetic and cellular resources to enable genotype-phenotype associations, it can be challenging to make such sensitive data available to the scientific community, as it often falls in the protected health information category, which, if shared, could compromise patient privacy and affect identifiability. Thus, the depth of shareable information must be appropriately balanced with donor privacy. Increasing diversity in cellular models also raises obvious questions of feasibility, as it requires laboratories to invest time and financial resources to incorporate additional hPSC lines into experimental paradigms. As discussed above, some laboratories may leverage diverse cell lines to interrogate known alleles across different genetic backgrounds using targeted approaches and thus require relatively small sample sets, while others may engage in discovery studies such as mapping the effects of genetic variants on cellular phenotypes which require substantial scale. Here, repositories with well-characterized, diverse and accessible hPSC lines, combined with additional support from research funding mechanisms specifically for purposes of incorporating hPSC lines from underrepresented populations, and clear reporting on ancestry selection in individual studies will be critical for the practical implementation of these resources (see additional recommendations from ref. 23).

As efforts are underway to increase diversity, it is worth taking a moment to consider how different populations are ascertained and described in hPSC collections. Regarding ascertainment, most cell banks use self-reported race or ethnicity as opposed to genetically inferred ancestry (with HipSci being a notable exception). Self-reported race or ethnicity reflects identity categories that can change over time, while genetically inferred ancestry (e.g., quantitative estimates of ancestral components by continent) reflects aspects of underlying biology which remain static for a given individual. Both types of data provide relevant information, but reliance upon self-reported race or ethnicity alone presents several specific limitations. The 2020 United States Census provides a timely example of how shifting social, political and cultural factors can influence self-reporting^24^, which is less reliable for populations composed of multiple ancestries and individuals who identify with multiple races or ethnicities^25^. Indeed, as discussed by ref. 26, an individual’s racial or ethnic identity may have little concordance with their genetic ancestry. One study investigating the accuracy of self-reporting for over 9000 individuals found that the method of data collection itself, in this case, a requisition form versus consultation, was sufficient to impact the level of concordance with genetic ancestry^27^. Another study analyzing nearly 2000 individuals in a pediatric HIV/AIDS cohort asked to self-identify as either “Black/African American”, “White” or “Hispanic”, found that when using the highest % genetic ancestry, 9.5% of subjects were mis-identified based on self-reporting and when $\ge$75% genetic ancestry of a specific population was required, 26% of individuals were mis-identified based on self-reporting^28^. These and other studies underscore how reliance upon self-reported race or ethnicity in hPSC collections may impact the accuracy as well as the longevity of the resources particularly as identity labels, some with fraught history, shift. Inclusion of genetically inferred ancestry is one strategy to improve the accuracy of cell-based resources, to achieve greater insight into the genetic architectures of specific subpopulations and to ensure that resources maintain their utility as identity labels change. Coupling this information with self-reported race or ethnicity using standardized nomenclature will provide a more complete picture of individual donors. This will of course require clear communication to ensure that donors understand and agree to genetic analyses to infer ancestry.

**Fig. 2:**
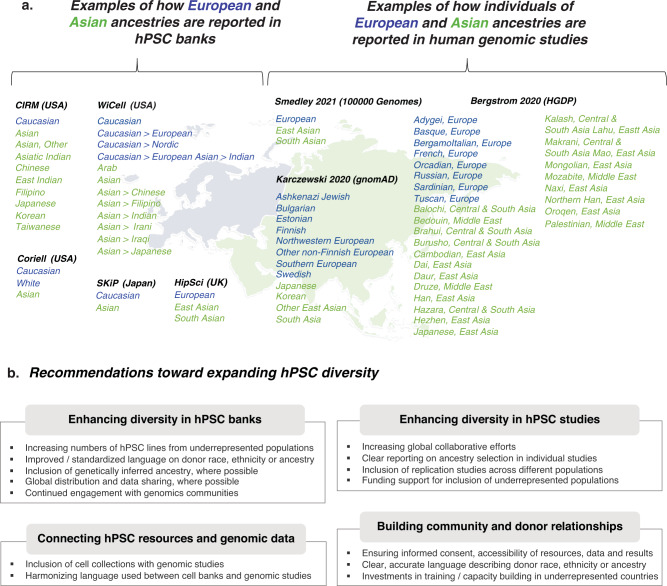
Considerations for reporting and expanding stem cell diversity. **a** Left, Examples of how individuals of European (blue) and Asian (green) ancestries are reported in current hPSC banks, including CIRM (USA), WiCell (USA), Coriell (USA), SKiP (Japan) and HipSci (UK). Right, Examples of how individuals of European (blue) and Asian (green) ancestries are reported in human genomic studies, including Bergstrom et al. 2020 (Human Genome Diversity Project (HGDP))^37^, Karczewski et al. 2020 (gnomAD)^38^ and Smedley et al. 2021 (100,000 Genomes Pilot)^39^. **b** Key recommendations toward expanding hPSC diversity. Map adapted from Templates by Yourfreetemplates.com/.

Regarding the language used to describe race, ethnicity and/or ancestry, there is a general lack of concordance between different hPSC banks, between different genomic studies, and across hPSC banks and genomic studies (Fig. 2a). Moreover, some hPSC banks rely on terms such as “Other” or “More than one race”, which fails to capture the increasing degree of ancestral complexity in global populations and essentially excludes these individuals from accurate representation. These issues make it challenging to identify relevant hPSC lines in order to pursue insights from human genomic datasets. As one example, the HLA-B*5701 variant associated with a hypersensitivity to Abacavir, a medication used to treat HIV, has a frequency of 13.6% among individuals in the Masai group in Kenya, 0% among individuals in the Yoruba group in Nigeria and 5.8% among individuals with European ancestry^29^. Here, the allelic variant does not segregate within the population terms used in any hPSC banks. While different studies will require varying levels of granularity in the populations under investigation, current estimates put the number of subcontinental ancestries at a minimum of 21, with 97.3% of individuals harboring ancestral heterogeneity^30^. In other words, while many hPSC collections were launched prior to or simultaneous with large-scale genomic initiatives (Fig. 1a), it is essential for hPSC collections to now consider how best to adapt to the rapidly expanding genomic insights from ancestrally diverse populations (Fig. 1c).

In a best-case scenario, for participants who have consented to iPSC derivation, material for iPSC reprogramming and banking would be collected alongside material for genomic and/or phenotypic studies, thus providing a direct link between these data and available cellular resources (Fig. 2b). Notably, such initiatives must be paired with community engagement efforts to ensure that participants have an appropriate understanding of how their samples will be used and stored, of the possible scientific and medical benefits that might ensue, but also that such benefits might not be immediate and/or personal. Efforts such as NeuroDev have succeeded in establishing sample collections for both exome sequencing as well as lymphocyte cell banking from individuals in South Africa^31^ and similar approaches could be undertaken for future hPSC collections. Working with participants from The Brazilian Longitudinal Study of Adult Health (ELSA-Brasil), laboratories within Brazil have derived iPSC lines and performed ancestry analyses to establish cellular resources that better reflect the Brazilian population, linked with clinical phenotyping data^32^. Alternatively, as groups like TOPMed^33^ and 1000 Genomes^34^ are expanding their reference populations, and cell banks like the California Institute for Regenerative Medicine are utilizing SNP microarrays to assay genomic integrity, these data could be combined to ascertain more refined ancestry predictions as opposed to relying solely on self-reported race or ethnicity. These more diverse reference panels will also further enable analyses of experimental models from underrepresented populations. At a minimum, standardized and more accurate descriptions of race, ethnicity, and ancestry should be used in future hPSC collections. Here, frameworks developed for reporting data in genomic studies could be leveraged to provide greater harmonization across disciplines (e.g., Morales 2018 Genome Biology)^35^.

## Outlook

Moving forward, increasing genetic diversity in hPSC collections and experimental paradigms has the potential to both accelerate basic biological discovery and facilitate more equitable distribution of scientific benefit. Beyond the discrete points discussed above, this will require a deliberate shift in the mentality investigators use to structure experiments and address the pressing scientific questions in their respective fields.

### Reporting summary

Further information on research design is available in the Nature Portfolio Reporting Summary linked to this article.

## Supplementary information

Reporting Summary

## Source data

Source Data
